# Treadmill exercise has minimal impact on obesogenic diet-related gut microbiome changes but alters adipose and hypothalamic gene expression in rats

**DOI:** 10.1186/s12986-020-00492-6

**Published:** 2020-08-18

**Authors:** Sarah-Jane Leigh, Nadeem O. Kaakoush, Rosa M. Escorihuela, R. Frederick Westbrook, Margaret J. Morris

**Affiliations:** 1 Department of Pharmacology, School of Medical Sciences, UNSW, Sydney, NSW 2052 Australia; 2 Departament de Psiquiatria i Medicina Legal, Institut de Neurociències, Universitat Autònoma de Barcelona, Barcelona, Spain; 3 School of Psychology, UNSW, Sydney, NSW 2052 Australia

**Keywords:** Obesity, Microbiome, Exercise, Hypothalamus, White adipose tissue, Cafeteria diet

## Abstract

**Background:**

Exercise has been extensively utilised as an effective therapy for overweight- and obesity-associated changes that are linked to health complications. Several preclinical rodent studies have shown that treadmill exercise alongside an unhealthy diet improves metabolic health and microbiome composition. Furthermore, chronic exercise has been shown to alter hypothalamic and adipose tissue gene expression in diet-induced obesity. However, limited work has investigated whether treadmill exercise commenced following exposure to an obesogenic diet is sufficient to alter microbiome composition and metabolic health.

**Methods:**

To address this gap in the literature, we fed rats a high-fat/high-sugar western-style cafeteria diet and assessed the effects of 4 weeks of treadmill exercise on adiposity, diet-induced gut dysbiosis, as well as hypothalamic and retroperitoneal white adipose tissue gene expression. Forty-eight male Sprague-Dawley rats were allocated to either regular chow or cafeteria diet and after 3 weeks half the rats on each diet were exposed to moderate treadmill exercise for 4 weeks while the remainder were exposed to a stationary treadmill.

**Results:**

Microbial species diversity was uniquely reduced in exercising chow-fed rats, while microbiome composition was only changed by cafeteria diet. Despite limited effects of exercise on overall microbiome composition, exercise increased inferred microbial functions involved in metabolism, reduced fat mass, and altered adipose and hypothalamic gene expression. After controlling for diet and exercise, adipose *Il6* expression and liver triglyceride concentrations were significantly associated with global microbiome composition.

**Conclusions:**

Moderate treadmill exercise induced subtle microbiome composition changes in chow-fed rats but did not overcome the microbiome changes induced by prolonged exposure to cafeteria diet. Predicted metabolic function of the gut microbiome was increased by exercise. The effects of exercise on the microbiome may be modulated by obesity severity. Future work should investigate whether exercise in combination with microbiome-modifying interventions can synergistically reduce diet- and obesity-associated comorbidities.

## Introduction

Overweight and obesity leads to reductions in physical and mental health, and quality of life [1], resulting in increased direct and indirect costs to the global economy [2]. Along with gross metabolic changes, obesity is associated with altered fecal microbial species diversity [3] and composition [4]. Separate studies involving transfer of obese human fecal microbiome samples induced fat gain in naïve mice [5] and supplementation with *Akkermansia muciniphila* improved insulin sensitivity and reduced body weight in overweight and obese people [6], providing some evidence for a potential role of diet- and obesity-associated gut microbiota changes in adiposity and metabolic dysfunction.

Weight loss through lifestyle intervention is an effective strategy for reducing obesity-related comorbidities [7]. One such intervention is moderate exercise, a practical and sustainable approach for people with overweight and obesity [8]: while exercising at this intensity is unlikely to cause weight loss independent of caloric restriction, it confers cardiovascular and metabolic benefits, and assists with weight maintenance [9, 10]. Furthermore, regular exercise is known to improve glucose regulation and insulin sensitivity [11] as well as reducing cardiovascular disease [12] and cancer risk [13], and there is increasing interest in the effects of exercise on the gut microbiota.

The first study to indicate an effect of exercise on fecal microbiome showed that elite, professional athletes exhibited a distinct microbiome composition with increased microbial species diversity [14]. However, since athletes consume a distinct diet from healthy people in the community, further work has been undertaken in rodents to identify the specific effects of exercise on fecal microbiome. A recent systematic review of primarily rodent studies concluded that while there was no consistent effect of exercise on microbial species richness, exercise increases the relative abundance of *Firmicutes* [15]. The different types of exercise used (forced versus voluntary) have been shown to exert different effects on fecal microbiome composition in mice [16, 17] which may in part explain the inconsistent findings.

Furthermore, there were considerable differences in exercise duration used (6–16 weeks) which may contribute to the range of responses observed. Microbiome compositional changes were observed in mice maintained on a healthy diet following 6 weeks of moderate treadmill exercise [16] and after 16 weeks in mice fed a high-fat diet [18]. In contrast, eight weeks of low-to-moderate exercise did not confer microbiome compositional changes in mice fed a high-fat diet [19]. Furthermore, most studies examining the effects of treadmill exercise on fecal microbiome in diet-induced obesity used a design where exercise was co-administered with high-fat diet, making it difficult to translate the findings to people, in terms of implementing exercise after a history of unhealthy eating and obesity.

Here we sought to examine whether moderate treadmill exercise in rats could exert benefits to gut microbiome composition following exposure to either a healthy or a high-fat, high-sugar western-style cafeteria diet. We aimed to investigate whether any changes in gut microbiome were associated with altered gene expression in white adipose tissue (WAT) and the hypothalamus, which are known to be affected by both obesogenic diets and exercise.

## Materials and methods

### Subjects and diet manipulation

This protocol was approved by the Animal Care and Ethics Committee of UNSW Sydney in accordance with the Australian guidelines for the use and care of animals for scientific purposes (National Health and Medical Research Council).

Forty-eight male Sprague-Dawley rats (6–7 weeks, 165-185 g; Animal Resource Centre, Australia) were housed 3/box (18-22 °C; 12 h light/dark) and handled daily for one week while maintained on standard chow (11 kJ/g; Premium Rat and Mouse Maintenance diet; Gordon’s Specialty Stock Feeds, Australia) and water ad libitum.

Following acclimatization, weight-matched groups were randomly allocated to Chow plus water or Cafeteria diet (*n* = 12 rats, *n* = 4 cages per group) ad libitum which comprised 10% sucrose solution alongside commercially-produced cakes, cookies and savoury foods [20] in addition to chow and water. Body weight and 24-h food intake were measured twice weekly and food intake was calculated assuming equal intake per rat in each cage. Body composition was measured during week 7 by EchoMRI-900 (EchoMRI LLC, USA).

### Treadmill exercise

Following 3 weeks, half the rats in each dietary condition were allocated to treadmill exercise (generating 4 weight-matched groups: Chow Sedentary (CSed), Chow Exercise (CEx), Cafeteria Sedentary (CafSed) and Cafeteria Exercise (CafEx)) for 4 weeks until the day before sacrifice. Moderate exercise consisted of 10-12 m/min for 45 min, five days a week at zero incline. The first week comprised two training sessions (0–10 m/min) after which time spent at 12 m/min was gradually increased. Sedentary rats were placed in stationary treadmills during the exercise protocol. Exercising rats were closely monitored. Three CafEx rats showed signs of fatigue, distress or vocalisation, and were removed from the treadmill and thereafter exercised at a lower intensity (5-6 m/min for 45 min). These rats were analysed together with moderately exercised CafEx rats.

### Sample collection

At 7 weeks of diet, rats were deeply anaesthetized (ketamine/xylazine 15/100 mg/kg intraperitoneally). Body weight, naso-anal length, girth and blood glucose were measured following induction of anesthesia. Rats were decapitated for trunk blood.

The hypothalamus (within coronal block defined by rostro-caudal limits of Circle of Willis) was rapidly dissected and collected. Retroperitoneal and gonadal WAT, and liver were dissected and weighed. One fecal pellet was removed from the distal colon. Tissue and feces were snap frozen in liquid nitrogen and stored at -80 °C.

### Protein and triglyceride measurements

Plasma leptin and insulin concentrations were measured using commercial kits (CAT#90040 and CAT#90060, CrystalChem Inc., USA). Plasma and liver triglyceride content were measured spectrophotometrically using triglyceride reagent (Roche Diagnostics Australia Pty Ltd., Australia) at 37 °C alongside a standard curve generated from glycerol standard (G7793-5ML, Sigma-Aldrich Pty Ltd., Australia). Livers were extracted by homogenization in 2:1 chloroform/methanol and incubated overnight; 0.6% NaCl was added and samples were vortexed and centrifuged (1000 g for 10 min). The lower phase was then evaporated at 40 °C under nitrogen gas. Dried extract was re-dissolved in absolute ethanol and measured spectrophotometrically. Retroperitoneal WAT IL-6 content was determined using a DuoSet IL-6 Rat ELISA (DY506, R&D Systems Inc.) following manufacturers’ recommendations.

### RNA extraction and gene expression assays

RNA was extracted from hypothalamus and retroperitoneal WAT using TRI Reagent (Sigma-Aldrich Pty Ltd., Australia). Following DNAse I treatment (Catalogue# 42885; Merck, Australia), 1.5 or 2 μg of RNA (WAT and hypothalamus respectively) were reverse transcribed to produce cDNA (High Capacity Reverse Transcriptase Kit; Thermofisher Scientific, USA). Gene expression was assessed using Taqman inventoried gene expression assays (Life Technologies Australia Pty Ltd., Australia; see Supplementary Table 1). Genes of interest were normalized against the geometric mean of the two most stable housekeeping genes (*Gapdh* and *Hprt1* for WAT, *Hprt1* and *B2m* for hypothalamus) identified by the Normfinder package [21]. Analysis of relative gene expression was performed using the ∆∆CT method normalized to an independent calibrator [22] made from all samples.

### Statistical analyses

Data were analyzed using two-way between-subjects ANOVA, while measures over time were analysed using 3-way mixed ANOVA. Post-hoc pairwise comparisons were performed using a Tukey adjustment where appropriate (THSD) and presented in the associated figures and tables only when *p* < 0.05. Pearson’s correlations were used to identify associations. All analyses were completed using IBM SPSS Statistics 23 (Australia).

### Fecal DNA extraction, microbiome community sequencing and statistical analyses

DNA extraction was performed using the PowerFecal DNA Isolation Kit (MoBio Laboratories, USA). Microbial community composition was assessed by Illumina amplicon sequencing (2 × 250 bp MiSeq chemistry, V4 region, 515F-806R primer pair) using a standard protocol. Sequence data were analyzed using Mothur [23] using modified commands from MiSeq SOP [24], including alignment with the SILVA database, singleton removal, chimera checking with UCHIME and classification against the latest RDP training set. Sequence depth was normalized by subsampling to 8346 total clean reads per sample.

Operational taxonomic unit (OTU) correlations were completed using Calypso [25], with the Benjamini-Hochberg false discovery rate (FDR) procedure [26] used to control for multiple tests. FDR-corrected DESeq2 was performed using the Phyloseq [27] R package for the negative binomial Walk test in DESeq2 [28]. OTU abundances were analyzed using SPSS with Kruskal-Wallis tests, followed by non-parametric Bonferroni-Dunn post-hoc testing where appropriate. OTUs of interest were identified using SINA Aligner [29].

Alpha diversity metrics, distanced-based linear modelling (dbLM), permutational multivariate ANOVA (PERMANOVA), non-metric multidimensional scaling (nMDS) and canonical analysis of principal coordinates were completed using Primer V6 (Primer-E Ltd., Plymouth, United Kingdom [30]). All Primer analyses utilized a Bray-Curtis similarity matrix constructed at the OTU level.

Phylogenetic Investigation of Communities by Reconstruction of Unobserved States (PICRUSt) was performed using Galaxy web to predict putative functions (through metagenomic prediction) from the 16S OTU data using Greengenes 13.5 for taxonomic classification [31]. Pathway counts were compared across groups using FDR-corrected Kruskal-Wallis tests followed by non-parametric Bonferroni-Dunn post-hoc testing where appropriate.

## Results

### Energy intake, body weight and composition, and WAT gene expression

**Fig. 1:**
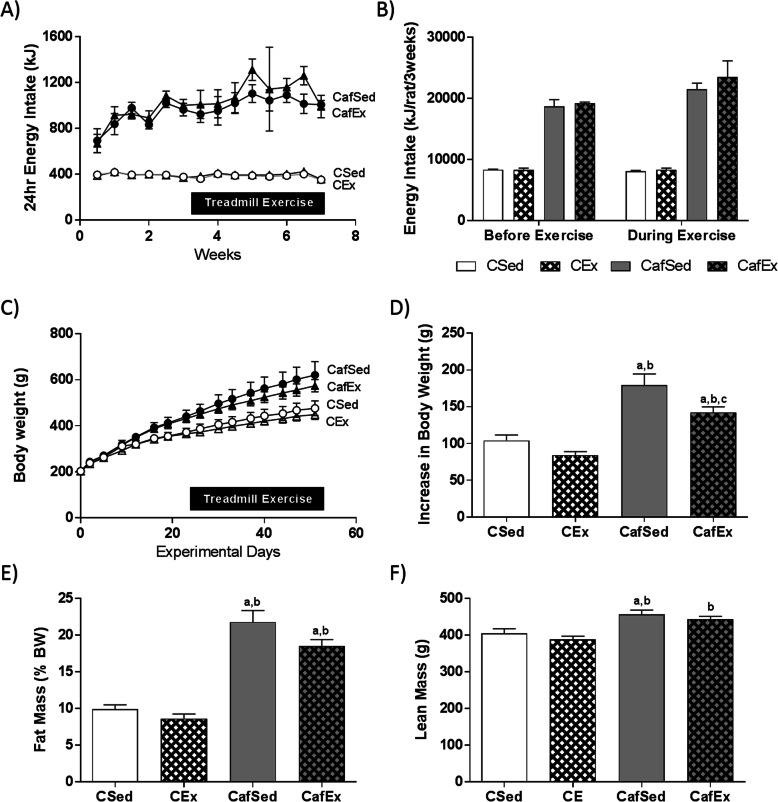
Short-term treadmill exercise reduces body weight gain and fat mass and alters WAT expression without affecting energy intake. **a** Energy intake over the study and **b** average weekly energy intake before and during treadmill exercise intervention. **c** Body weight of the study and **d** body weight gain during treadmill exercise intervention. **e** Fat mass (as a percentage of body weight) and **f** absolute lean mass; Data expressed as mean ± SEM; *n* = 4 for cage data, *n* = 11–12 for individual data; data were analyzed by two-way ANOVA followed by Tukey-adjusted post-hoc testing. ^a^*p* < 0.05 relative to CSed, ^b^*p* < 0.05 relative to CEx, ^c^*p* < 0.05 relative to CafSed

Over the 7-week study, cafeteria-fed rats ate more than twice the energy consumed by chow-fed groups (Fig. 1a; CSed: 18990 kJ/rat; CEx: 19380 kJ/rat; CafSed: 49234 kJ/rat; CafEx: 53485 kJ/rat). When energy intake was stratified into pre- and during-exercise intervention, a significant interaction between time and diet was observed (Fig. 1b; F (1, 12)=9.42, *p* = 0.01) which appeared to be due to increased cafeteria diet intake while rats were exercising, although this comparison did not reach statistical significance. Significant interactions between time and diet were also identified for fat and carbohydrate intakes (Supplementary Figure 1A; F (1, 12)=5.92, *p* = 0.032 and Supplementary Figure 1C; F (1, 12)=6.83, *p* = 0.023 respectively) where both macronutrient intakes increased in the cafeteria-fed rats during exercise. Protein intake, while unaffected by time or exercise, was elevated by cafeteria diet (Supplementary Figure 1B; F (3, 12)=24.06, *p* < 0.001). Sucrose intake increased over time (Supplementary Figure 1D; F (1, 6)=51.90, *p* < 0.001) but was not affected by exercise.

All rats gained body weight over time (Fig. 1c) and cafeteria-fed rats gained significantly more than chow-fed controls prior to (F (1, 44)=61.54, *p* < 0.001) and during (Fig. 1d; F (1, 44)=57.70, *p* < 0.001) exercise. Exercise significantly reduced weight gain overall (F (1, 44)=8.97, *p* = 0.004). THSD post-hoc comparisons showed that compared to CSed and CEx, CafSed (*p* < 0.001 and *p* < 0.001) and CafEx (*p* = 0.038 and *p* < 0.001) gained significantly more weight over the exercise intervention. Of note, CafSed rats gained significantly more weight than CafEx rats over the exercise intervention (*p* = 0.043).

Body composition data following 4 weeks of treadmill exercise showed that relative fat mass was significantly increased by cafeteria diet (Fig. 1e; F (1, 44)=154.58, *p* < 0.001; absolute fat mass presented in Table 1) and reduced by exercise (F (1, 44)=6.35, *p* = 0.015). THSD post-hoc comparisons revealed that relative fat mass in both cafeteria-fed groups were significantly greater than both chow-fed groups. Lean mass however showed only an overall diet effect (Fig. 1f; F (1, 44)=27.34, *p* < 0.001) with significantly more lean mass in cafeteria-fed rats than chow-fed rats.

**Table 1:** Anthropometric measures at tissue collection and plasma measures

Measure	CSed	CEx	CafSed	CafEx	Main Effects (*p*-value)		
					Diet	Exercise	Interaction
Terminal Body Weight (g)	475.4 ± 14.9	455.6 ± 31.4	625.5 ± 26.2^a,b^	583.3 ± 12.4^a,b^	**< 0.001**	0.073	0.512
Nasoanal Length (cm)	24.5 ± 0.2	24.4 ± 0.2	25.0 ± 0.2	25.0 ± 0.1^b^	**< 0.001**	0.914	0.450
Girth (cm)	19.1 ± 0.2	18.9 ± 0.2	22.3 ± 0.6^a,b^	21.5 ± 0.4^a,b^	**< 0.001**	0.186	0.505
Liver score	0.17 ± 0.09	0.17 ± 0.09	1.96 ± 0.17^a,b^	2.08 ± 0.22^a,b^			**< 0.001**
Heart Weight (g)	1.39 ± 0.04	1.34 ± 0.03	1.69 ± 0.07^a,b^	1.62 ± 0.03^a,b^	**< 0.001**	0.170	0.884
Absolute fat mass (g; EchoMRI)	47.51 ± 3.63	38.78 ± 3.09	139.23 ± 16.27^a,b^	107.35 ± 6.81^a,b^	**< 0.001**	**0.031**	0.212
RP Fat Pad Weight (% BW)	2.22 ± 0.16	1.87 ± 0.15	5.87 ± 0.43^a,b^	4.73 ± 0.47^a,b^	**< 0.001**	**0.022**	0.142
Epidydimal Fat Pad Weight (% BW)	2.35 ± 0.14	2.12 ± 0.17	6.01 ± 0.54^a,b^	4.91 ± 0.21^a,b^	**< 0.001**	**0.032**	0.126
Blood Glucose (mmol/L)	9.0 ± 0.4	9.7 ± 0.9	10.4 ± 0.5	10.2 ± 0.5	0.548	0.225	0.111
Plasma Insulin (ng/mL)	0.53 ± 0.12	0.27 ± 0.07	2.09 ± 0.32^a,b^	1.68 ± 0.43^a,b^	**< 0.001**	0.246	0.797
Plasma Leptin (ng/mL)	4.72 ± 0.55	3.81 ± 0.34	18.64 ± 1.43^a,b^	15.21 ± 1.21^a,b^	**< 0.001**	**0.037**	0.220
Plasma Triglycerides (mmol/L)	1.16 ± 0.12	0.86 ± 0.08	3.52 ± 0.32^a,b^	3.27 ± 0.25^a,b^	**< 0.001**	0.056	0.226
Liver Triglycerides (mg/g tissue)	3.77 ± 0.49	3.65 ± 0.44	23.32 ± 2.52^a,b^	20.58 ± 2.41^a,b^	**< 0.001**	0.423	0.465

Blood and plasma measures performed unfasted. Data expressed as mean ± SEM; *n* = 10–12. Data were analyzed using two-way ANOVA, followed by post-hoc multiple comparisons with a Tukey HSD correction. Liver score was analysed using a Kruskal-Wallis test followed by non-parametric Bonferroni-Dunn post-hoc testing

^a^*p* < 0.05 relative to CSed, ^b^p < 0.05 relative to CEx

Cafeteria diet also increased naso-anal length, girth, plasma insulin and triglycerides, with no effect of exercise (Table 1). Plasma leptin levels were significantly increased by diet (F (1, 43)=185.1, *p* < 0.001) and reduced by exercise (F (1, 43)=4.64, *p* = 0.037). Unfasted blood glucose did not differ between groups (Table 1).

In line with plasma leptin, retroperitoneal and epidydimal fat pad weights were significantly greater in cafeteria- than chow-fed rats (F (1, 44)=90.21, *p* < 0.001 and F (1, 44)=161.41, *p* < 0.001 for retroperitoneal and epidydimal fat pads respectively), and were significantly reduced by exercise overall (F (1, 44)=5.73, *p* = 0.021 and F (1, 44)=4.92, *p* = 0.032 for retroperitoneal and epidydimal fat pads respectively) (See Table 1).

### Microbial species diversity and microbiome composition

**Fig. 2:**
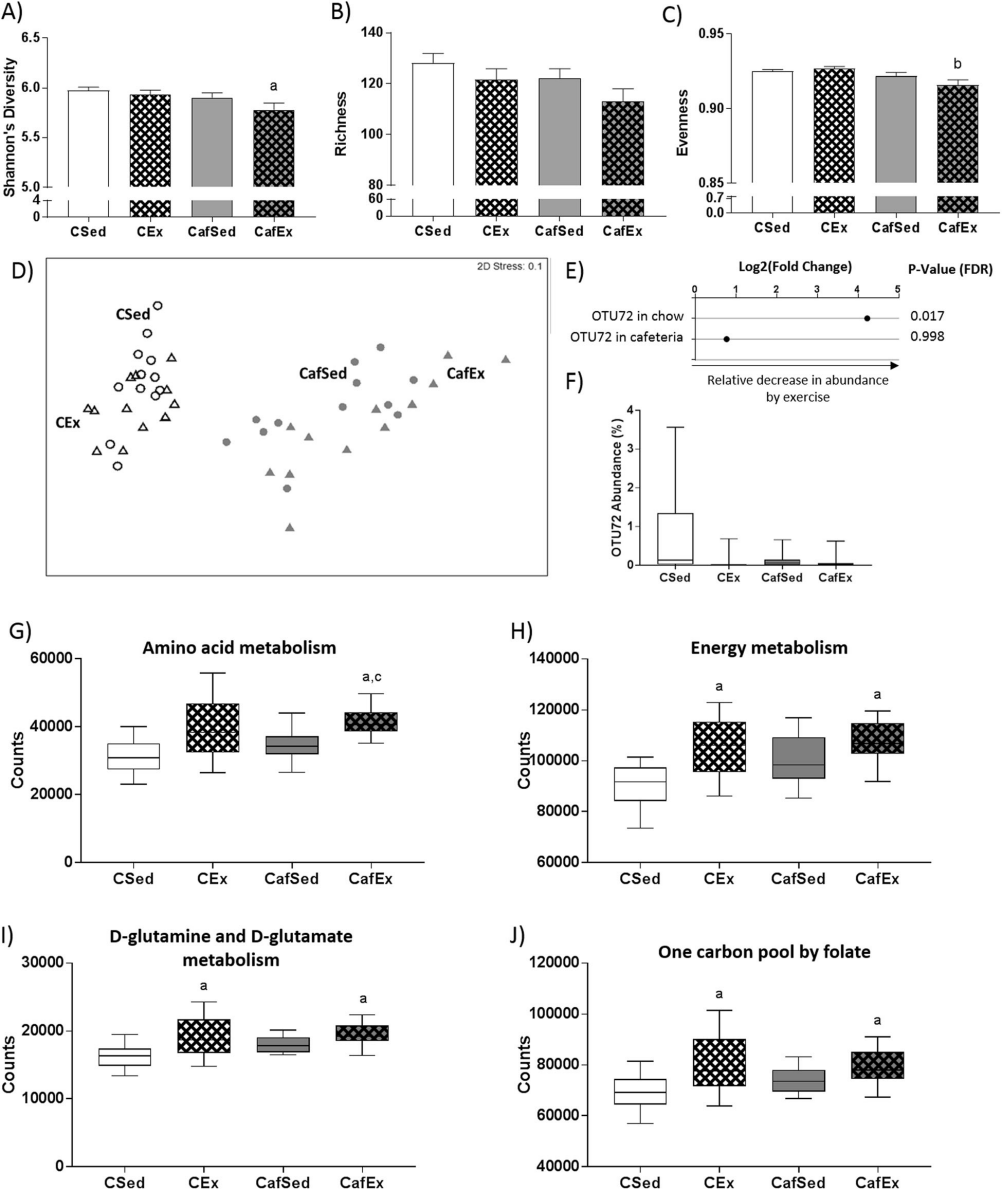
Impact of cafeteria diet and treadmill exercise intervention on fecal microbiota and inferred microbiome function at 7 weeks. **a** Shannon’s diversity, **b** microbial species richness and **c** evenness. Data expressed as mean ± SEM; *n* = 11–12; data were analyzed by two-way ANOVA followed by Tukey-adjusted post-hoc testing. **d** Non-metric multidimensional scaling (Bray-Curtis, 1000 permutations) showing similarity between fecal microbiota samples at 7 weeks. **e** *Muribaculum*_OTU72 identified by DESeq2 (adjusted p < 0.05) as differentially abundant with exercise in chow-fed rats. **f** Relative abundance of OTU72 at 7 weeks. Data expressed as box-and-whisker plots (min, IQR, max); *n* = 11–12; data were analyzed using Kruskal-Wallis test followed by non-parametric Dunn-Bonferroni post-hoc testing. **g** Amino acid metabolism, **h** overall energy metabolism, **i** D-glutamine and D-glutamate metabolism and **j** one carbon pool by folate predicted using PICRUSt from fecal microbiome data at 7 weeks. Data are expressed as box-and-whisker plots (min, IQR, max); *n* = 11–12; were analyzed by Kruskal-Wallis tests (FDR-adjusted overall p-value to account for multiple relevant pathways included in analysis) followed by non-parametric Bonferroni-Dunn post-hoc comparisons. Post-hoc symbols: ^a^*p* < 0.05 relative to CSed, ^b^*p* < 0.05 relative to CEx, ^c^*p* < 0.05 relative to CafSed

Microbial species diversity was assessed using Shannon’s diversity index, microbial species richness and microbial species evenness. Shannon’s diversity and evenness were significantly reduced by cafeteria diet overall (F (1, 44)=5.202, *p* = 0.027; Fig. 2a and f (1, 44)=8.278, *p* = 0.006; Fig. 2c respectively) and THSD post-hoc comparisons showed that CafEx rats exhibited reduced evenness relative to CEx (*p* = 0.017) and reduced Shannon’s diversity relative to CSed (*p* = 0.040). No significant differences were observed for bacterial species richness (Fig. 2b).

Microbiome composition at the OTU level was significantly affected by diet (Pseudo-F (1, 32)=12.17, *p* = 0.030) and cage (Pseudo-F (3, 32)=1.55, *p* = 0.006) but not by exercise when assessed using 4-way PERMANOVA (999 permutations) and confirmed with non-metric multidimensional scaling (Fig. 2d). Supplementary Figure 2 shows groups differences in microbiome composition at the Phylum level.

DESeq2 analyses were used to identify OTUs differentially enriched with exercise exposure amongst the top 200 OTUs. While exercise was not associated with differentially expressed OTUs in cafeteria-fed rats, *Muribaculum*_OTU72 was significantly depleted in CEx rats relative to CSed (Fig. 2e, relative abundance in Fig. 2f; this OTU was originally classified as *Akkermansia* when aligned with the RDP reference library). *Muribaculum*_OTU72 was putatively identified as an unknown bacterium from the genus *Muribaculum* using SINA Aligner (97.6% alignment identity).

### Predicted microbiome function

To determine whether the subtle microbiome composition changes observed with exercise affected microbiome function, we inferred microbiome function using PICRUSt. Following an FDR correction, amino acid metabolism (Fig. 2g; H (4)=16.3, *p* = 0.001), overall energy metabolism (Fig. 2h; H (4)=12.64, *p* = 0.006), D-glutamine and D-glutamate metabolism (Fig. 2i; H (4)=14.58, *p* = 0.002) and one carbon pool by folate (Fig. 2j; H (4)=11.44, *p* = 0.010) exhibited overall group differences. Amino acid metabolism was significantly elevated in CafEx relative to CSed (*p* < 0.001) and CafSed (*p* = 0.023) rats while the 3 other processes were significantly elevated in both exercised groups relative to CSed.

### WAT and hypothalamic gene expression

**Fig. 3:**
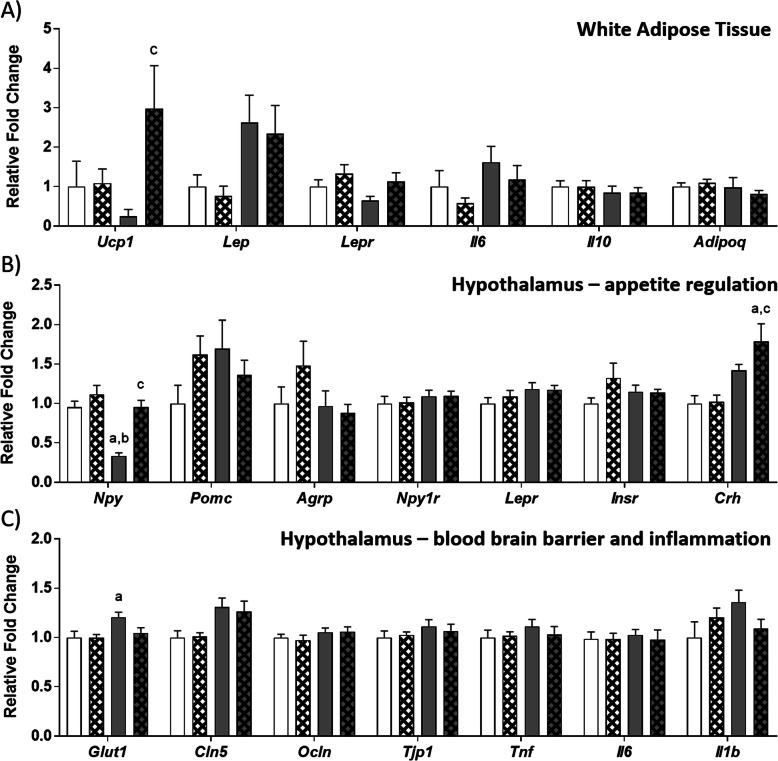
Gene expression in retroperitoneal WAT, hypothalamus and associations with gut microbiome changes. **a** Adipokine, metabolic and inflammatory gene expression in retroperitoneal WAT. **b** Feeding- and stress-related gene expression in the hypothalamus. **c** Blood-brain barrier and pro-inflammatory gene expression in the hypothalamus. *Adipoq*: adiponectin, *Agrp*: Agouti-related protein, *Cln5*: Claudin-5, *Crh*: Corticotrophin releasing hormone, *Glut1*: Glucose transporter 1, *Il6*: interleukin 6, *Il10*: interleukin-10, *Il1b*: interleukin 1 beta, *Insr*: Insulin receptor, *Lep*: Leptin, *Lepr*: Leptin receptor, *Npy*: Neuropeptide y, *Npy1r*: Neuropeptide y receptor 1, *Ocln*: Occludin, *Pomc*: Pro-opiomelanocortin, *Tjp1*: Tight junction protein 1, *Tnf*: Tumour necrosis factor, *Ucp1*: uncoupling protein 1; Data expressed as mean ± SEM; n = 11–12; data were analyzed by two-way ANOVA followed by Tukey-adjusted post-hoc testing. ^a^*p* < 0.05 relative to CSed, ^b^*p* < 0.05 relative to CEx, ^c^*p* < 0.05 relative to CafSed

Examination of WAT inflammatory signaling and browning genes (Fig. 3a) revealed a significant interaction effect for *Ucp1* (F (1, 40)=4.41, *p* = 0.042) while THSD post-hoc comparisons showed that CafEx rats exhibited elevated *Ucp1* expression relative to CafSed (*p* = 0.024). *Lep* expression was significantly elevated by cafeteria diet consumption (F (1, 41)=4.98, *p* = 0.031), while *Lepr* expression was increased with exercise overall (F (1, 41)=4.75, *p* = 0.035). No significant differences were observed in pro-inflammatory markers or *Adipoq* gene expression.

Hypothalamic gene expression was analyzed to determine if 4 weeks of moderate exercise was sufficient to reverse diet-induced changes in expression of genes involved in feeding (Fig. 3b), blood brain barrier integrity and pro-inflammatory signaling (Fig. 3c). A significant interaction effect was observed for *Npy* gene expression in the hypothalamus (F (1, 41)=7.58, *p* = 0.009), and THSD showed that CafSed rats exhibited downregulated *Npy* relative to CSed (*p* < 0.001), CEx (*p* < 0.001) and CafEx rats (*p* < 0.001). No significant differences were observed for *Pomc*, *Agrp*, *Npy1r*, *Lepr* or *Insr*. *Crh* gene expression was significantly increased in cafeteria-fed rats overall (F (1, 41)=14.89, *p* < 0.001), but was unaffected by exercise (F (1, 41)=1.06, *p* = 0.309). Post-hoc THSD analysis revealed that CafEx rats exhibited significantly upregulated *Crh* relative to CSed (*p* = 0.001) and CEx (*p* = 0.001).

Both *Cln5* and *Glut1* were significantly increased in cafeteria-fed rats overall (F (1, 41)=10.96, *p* = 0.002 and F (1, 41)=5.44, *p* = 0.025 respectively). While no group differences were apparent for *Cln5* using THSD comparisons, *Glut1* expression was significantly elevated in CafSed rats relative to CSed controls (*p* = 0.045). No significant differences were observed in *Ocln, Tjp1* or any of the pro-inflammatory genes assessed.

### Associations between variables of interest and microbiome

When variables were assessed for their unique contribution to the variance in overall microbiome composition, several adiposity measures, as well as retroperitoneal WAT *Il6* gene expression and hypothalamic *Crh* and *Npy* expression, were identified as significant predictors of global microbiome composition (Supplementary Table 2). When the contribution of variables of interest to overall microbiome composition was assessed while controlling for diet and treadmill exercise, both liver triglyceride concentration (R^2^ = 0.031, *p* = 0.008) and retroperitoneal WAT *Il6* gene expression (R^2^ = 0.026, *p* = 0.027) were significant predictors of microbiome composition at the OTU level (Table 2, complete model predicts 34.7% of the variance in microbiome composition).

**Table 2:** The shared contributions of diet, exercise and variables of biological relevance on the variance observed in microbiome composition using distance-based linear modelling

Variable	SS	Pseudo-F	*P*-Value	R^2^	Cumulative R^2^
Diet	18,456	14.502	**0.001**	0.266	0.266
Exercise	1686.7	1.337	0.073	0.024	0.290
Liver triglyceride content	2157.7	1.742	**0.008**	0.031	0.321
WAT Il6 expression	1822	1.490	**0.027**	0.026	0.347

Sequential multiple regression (captured by the Bray-Curtis similarity matrix at the OTU level, max 1000 permutations) involves interrogating the conditional contribution of each variable in order of entry into the model (to determine whether variables contribute significantly to the variance explained in the presence of other variables); here, diet and exercise conditions were added before any metabolic predictors were considered and the final model containing only statistically significant covariates is shown. Metabolic predictors included in the sequential regression were selected based on their predictive value, while trying to eliminate variables with high covariance; *N* = 42–46

**Fig. 4:**
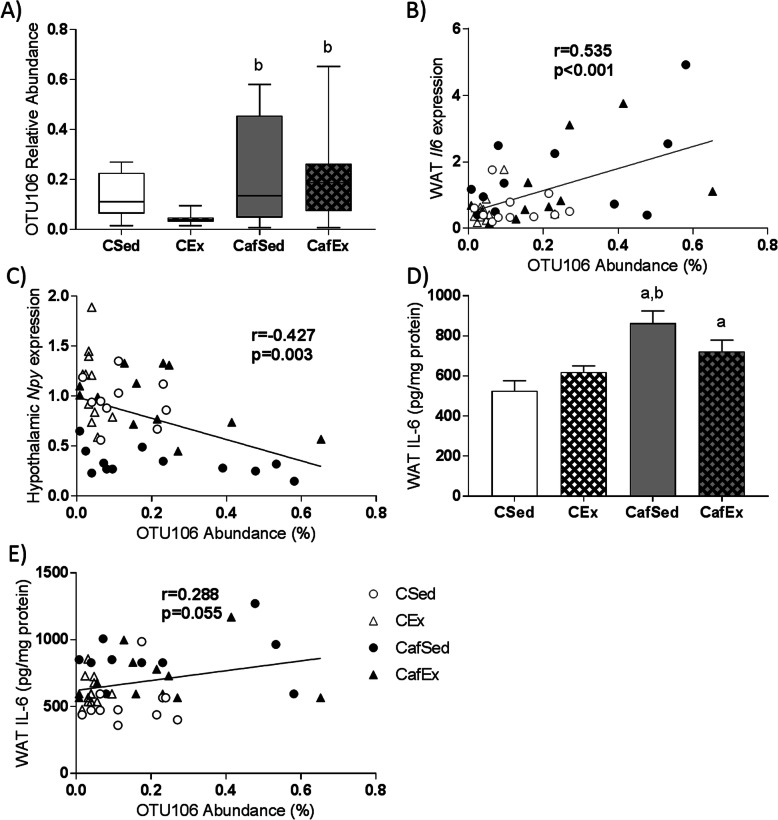
Relationship between OTU106, and hypothalamic *Npy* and WAT IL-6 expression (**a**) Relative abundance of OTU106; data expressed as box-and-whisker plots (min, IQR, max); n = 11–12; data were analyzed using Kruskal-Wallis test followed by non-parametric Dunn-Bonferroni post-hoc testing. Scatterplots for OTU106 abundance and (**b**) *Il6* gene expression in WAT, and (**c**) *Npy* gene expression in the hypothalamus, showing overall lines of best fit; *n* = 46. **d** WAT IL-6 protein content; data expressed as mean ± SEM; *n* = 10–12; data were analyzed by two-way ANOVA followed by Tukey-adjusted post-hoc testing. **e** Scatterplot for OTU106 abundance and IL-6 protein content in WAT, showing overall lines of best fit; *n* = 45. ^a^*p* < 0.05 relative to CSed, ^b^*p* < 0.05 relative to CEx, ^c^*p* < 0.05 relative to CafSed

*Porphyromonadaceae unclassified*_OTU106 was increased with cafeteria diet (Fig. 4a; H (4)=11.42, *p* = 0.010) and significantly associated with WAT *Il6* gene expression (Fig. 4b). Interestingly, this OTU was also negatively correlated with hypothalamic *Npy* gene expression (Fig. 4c). IL-6 protein in WAT exhibited a significant interaction effect (F (1, 41)=5.310, *p* = 0.026; Fig. 4d) and was positively associated with *Porphyromonadaceae unclassified*_OTU106 (Fig. 4e), following a similar, although non-significant, trend to that observed with *Il6* gene expression. Like Il6 gene expression, WAT IL-6 content was a significant independent predictor of overall microbiome composition (Supplementary Table 2) but was not a significant predictor in the final model, after controlling for diet and exercise. SINA Aligner putatively identified *Porphyromonadaceae unclassified*_OTU106 as a strain of *Bacteroides eggerthii*, a Gram-negative bacterium known to hydrolyze carbohydrates including simple sugars [32].

## Discussion

We found that 4 weeks of moderate treadmill exercise reduced fat mass and plasma leptin concentrations and altered WAT expression of some adipokine- and metabolism-associated genes. Overall microbiome composition and microbial species diversity was changed by cafeteria diet but not by exercise. However, predicted microbial functions associated with metabolism were increased by exercise. Cafeteria diet-induced changes in hypothalamic *Npy* and *Glut1* gene expression were returned to control levels by exercise.

Exercise induced modest changes in gut microbiome composition that were statistically significant in chow-fed rats only and the relative abundance of OTU72 was significantly reduced in chow-fed rats that exercised (CEx) rats relative to sedentary controls (CSed). While CafEx rats exhibited reduced microbial species diversity, this reduction appeared dependent on cafeteria diet exposure rather than exercise. To date work examining the effects of exercise on microbial species diversity has produced inconsistent results [15]: some rodent studies have shown that exercise is associated with reductions in fecal microbial species richness [16] while others have reported no such effect [33].

After 4 weeks of treadmill exercise we did not observe changes in overall microbiome composition. Our data are in line with findings in humans showing that changes in microbiome composition are dependent on obesity status, such that more severe obesity is associated with smaller effects of exercise [34]. Since the cafeteria diet used here [20] tends to produce a more severe metabolic phenotype than purified high-fat diets [35–38], which to our knowledge have been used in all studies investigating the interrelationship between exercise and diet on microbiome composition, the diet-induced effects on microbiome composition here may be more resistant to the effects of exercise than previously reported. Additionally, a number of rodent studies report no differences in overall microbiome composition with exercise [17, 19], and there is evidence that this effect may be moderated by age [39] which may have contributed to the inconsistent findings in the literature.

Predicted microbial functions associated with metabolism, specifically overall energy metabolism, amino acid metabolism, one carbon pool by folate and D-glutamine and D-glutamate metabolism, were increased in exercised rats. This is in line with metagenomic results where fecal microbiome from male elite athletes exhibited increased amino acid biosynthesis and overall energy metabolism relative to sedentary, normal-weight controls [40]. While PICRUSt analysis produces predicted functional data, unlike metagenomic analysis, this is an interesting finding that warrants follow-up to determine if and how exercise shifts the metabolic profile of the gut microbiome and whether any such shift is affected by exercise intensity and duration. Furthermore, confirming these results across a range of diets would be useful to determine whether the shift in microbial function with exercise is modulated by the macro- and micronutrients available.

Here, a moderate exercise intervention reduced fat mass and plasma leptin concentrations, and increased WAT *Lepr* gene expression in both exercised groups and *Ucp1* gene expression in exercised, cafeteria-fed (CafEx) rats uniquely. Increased *Ucp1* in WAT depots is a marker of adipocyte beiging [41], known to be promoted by exercise [42], and is most likely related to exercise-induced fat loss. While there were no significant effects of exercise on WAT pro-inflammatory gene expression, after controlling for diet and exercise WAT *Il6* expression was significantly associated with global microbiome composition.

WAT is one of the major sources of IL-6 in obese humans [43] and mice [44, 45], which is a key component of the low-grade systemic inflammation observed in overweight and obesity [46] and is associated with insulin resistance [47]. WAT *Il6* expression was strongly associated with the relative abundance of a strain of *Bacteroides eggerthii* (OTU106), a Gram-negative sugar-scavenging bacterium [32] which is enriched in obese children relative to normal-weight controls [48]. The associations between WAT *Il6* expression, global microbiome composition and OTU106 abundance are therefore likely to be due to the effects of cafeteria diet on both adipose inflammatory processes and the gut microbiome. However, probiotic treatment with a strain of *Bifidobacterium* in mice fed a high-fat diet reduced WAT macrophage infiltration and plasma IL-6 concentration [49], indicating that changes to the gut microbiome may contribute to WAT inflammatory signaling and IL-6 production. Further studies determining whether specific bacterial species can modulate WAT IL-6 production are warranted, as interventions that could reduce WAT IL-6 expression in obesity may provide an avenue for preventing insulin resistance and type 2 diabetes.

In contrast to the overall effect of exercise on WAT gene expression, hypothalamic genes disrupted by cafeteria diet were typically normalized with exercise, with no differences observed in chow-fed rats. *Npy* was downregulated in CafSed rats, as shown previously [50], but normalized to control levels with exercise, which is in line with other rodent work showing increased hypothalamic NPY mRNA and protein in response to both acute and chronic exercise [51–54]. Hypothalamic *Glut1* gene expression was increased in CafSed rats and reduced to control levels with exercise. This is in contrast to studies showing that acute exercise increased GLUT1 protein expression across the rat brain [55] and prolonged exercise increases whole brain resting glucose uptake in people [56]. Further work investigating acute and chronic exercise-induced changes in *Glut1* expression in the hypothalamus and other brain regions is required.

Cafeteria diet-induced *Crh* upregulation in the hypothalamus was not normalized by exercise. *Crh* is transcribed to corticotrophin-releasing hormone (CRH), which activates the hypothalamic-pituitary-adrenal (HPA) axis, key in physiological and behavioral responses to stress. Involuntary exercise is a known stressor [57], and forced exercise increased activity in CRH neurons relative to both voluntary exercise and sedentary control groups in rats [58].

Exercise can impact the gut microbiome via several pathways, and there are multiple potential mechanisms by which these gut microbiome changes could subsequently confer health benefits. Exercise may shape microbiome composition and function by increasing host metabolic requirements and shifting metabolite availability. A recent study showed that marathon runners exhibit increased abundance of *Veillonella atypica*, which rely on lactate as a source of carbon, and inoculation with this species increased running time in mice [59]. Alternatively, exercise is known to activate the HPA axis [60]. The HPA axis appears to exert effects on the gut microbiome, and may be impacted by microbiome modification [61]. Therefore, the effects of exercise on stress behaviors and physiology may be in part due to exercise-induced changes to the gut microbiome, or vice versa.

## Conclusions

In summary, short-term (4 weeks) treadmill exercise induced subtle microbiome composition changes in chow-fed rats but did not overcome the microbiome changes induced by cafeteria diet. Despite this, predicted microbiome function was altered by exercise in both chow- and cafeteria-fed groups. Exercise reduced adiposity and altered both WAT and hypothalamic gene expression. WAT *Il6* expression was significantly associated with microbiome composition and negatively correlated with the abundance of a strain of *Bacteroides eggerthii*, bacteria known to scavenge sugars. Future work identifying the circumstances where exercise exerts effects on the microbiome, and the potential causal role of these changes for reducing diet- and obesity-associated comorbidities, is warranted.

## Supplementary information

**Additional file 1: Supplementary Table 1.** Taq assay probe information. **Supplementary Figure 1.** Weekly macronutrient intake and sucrose intake over the study. **Supplementary Figure 2.** Average composition of phyla between experimental groups. **Supplementary Table 2.** Distance-based linear modelling to determine contributions of metabolic and gene expression measures to the variance observed in microbiota composition at the OTU level.

## Data Availability

The study metadata and sequence data are available in the European Nucleotide Archive under accession number PRJEB36541. The study is currently set on private and will be released upon acceptance. All other data will be made available from the corresponding author on reasonable request.

## Abbreviations

*Adipoq*: Adiponectin
*Agrp*: Agouti-related peptide
*B2m*: Beta-2-microglobin
CEx: Chow Exercise
*Cln5*: Claudin 5
*Crh*: Corticotrophin-releasing hormone
CSed: Chow Sedentary
CafEx: Cafeteria Exercise
CafSed: Cafeteria Sedentary
dbLM: Distance-based linear modelling
FDR: False discovery rate
*Gapdh*: Glyceraldehyde 3-phosphate dehydrogenase
*Glut1*: Glucose transporter 1
*Hprt1*: Hypoxanthine phosephoribosyltransferase 1
*Il6*: Interleukin-6
*Insr*: Insulin receptor
*Lep*: Leptin
*Lepr*: Leptin receptor
nMDS: Non-metric multidimensional scaling
*Npy*: Neuropeptide Y
*Npy1r*: Neuropeptide Y receptor 1
*Ocln*: Occludin
OTU: Operational taxonomic unit
PERMANOVA: Permutational multivariate ANOVA
PICRUSt: Phylogenetic investigation of communities by reconstruction of unobserved states
*Pomc*: Pro-opiomelancortin
*Tjp1*: Tight junction protein 1
THSD: Tukey honest significant difference
WAT: White adipose tissue
*Ucp1*: Uncoupling protein 1
