# Supplementary material for: Treadmill exercise has minimal impact on obesogenic diet-related gut microbiome changes but alters adipose and hypothalamic gene expression in rats
Source: Nutr Metab (Lond). 2020 Aug 18;17:71. doi: 10.1186/s12986-020-00492-6 (PMC7437044; doi:10.1186/s12986-020-00492-6)
Supplement: Supplementary file 1 — Additional file 1: Supplementary Table 1. Taq assay probe information. Supplementary Figure 1. Weekly macronutrient intake and sucrose intake over the study. Supplementary Figure 2. Average composition of phyla between experimental groups. Supplementary Table 2. Distance-based linear modelling to determine contributions of metabolic and gene expression measures to the variance observed in microbiota composition at the OTU level. [file 12986_2020_492_MOESM1_ESM.docx]

**Supplementary Figures and Tables**

**Supplementary Table 1.** Taq assay probe information

| Gene name (abbreviation) | Unique assay identifier |
| --- | --- |
| Agouti-related protein (*Agrp*) | Rn01431703_g1 |
| Beta-2-microglobin (*B2m*) | Rn00560865_m1 |
| Claudin-5 (*Cldn5*) | Rn01753146_s1 |
| Corticotrophin releasing hormone (*Crh*) | Rn01462137_m1 |
| Glyceraldehyde 3-phosphate dehydrogenase (*Gapdh*) | Rn01749022_g1 |
| Glucose transporter 1 (*Glut1*) | Rn01417099_m1 |
| Hypoxanthine phosephoribosyltransferase 1 (*Hprt1*) | Rn01527840_m1 |
| Insulin receptor (*Insr*) | Rn00690703_m1 |
| Interleukin-1 beta (*Il1B*) | Rn00580432_m1 |
| Interleukin-6 (*Il6*) | Rn01410330_m1 |
| Interleukin-10 (*Il10*) | Rn00563409_m1 |
| Leptin (*Lep*) | Rn00565158_m1 |
| Leptin receptor (*Lepr*) | Rn01433205_m1 |
| Neuropeptide Y (*Npy*) | Rn01410145_m1 |
| Neuropeptide Y receptor 1 (*Npy1r*) | Rn02769337_s1 |
| Occludin (*Ocln*) | Rn00580064_m1 |
| Pro-opiomelanocortin (*Pomc*) | Rn00595020_m1 |
| Tumour necrosis factor alpha (*Tnf*) | Rn99999017_m1 |
| Uncoupling protein 1 (*Ucp1*) | Rn00562126_m1 |

Taq assays and their unique identifiers assessed in the hypothalamus and retroperitoneal white adipose tissue.


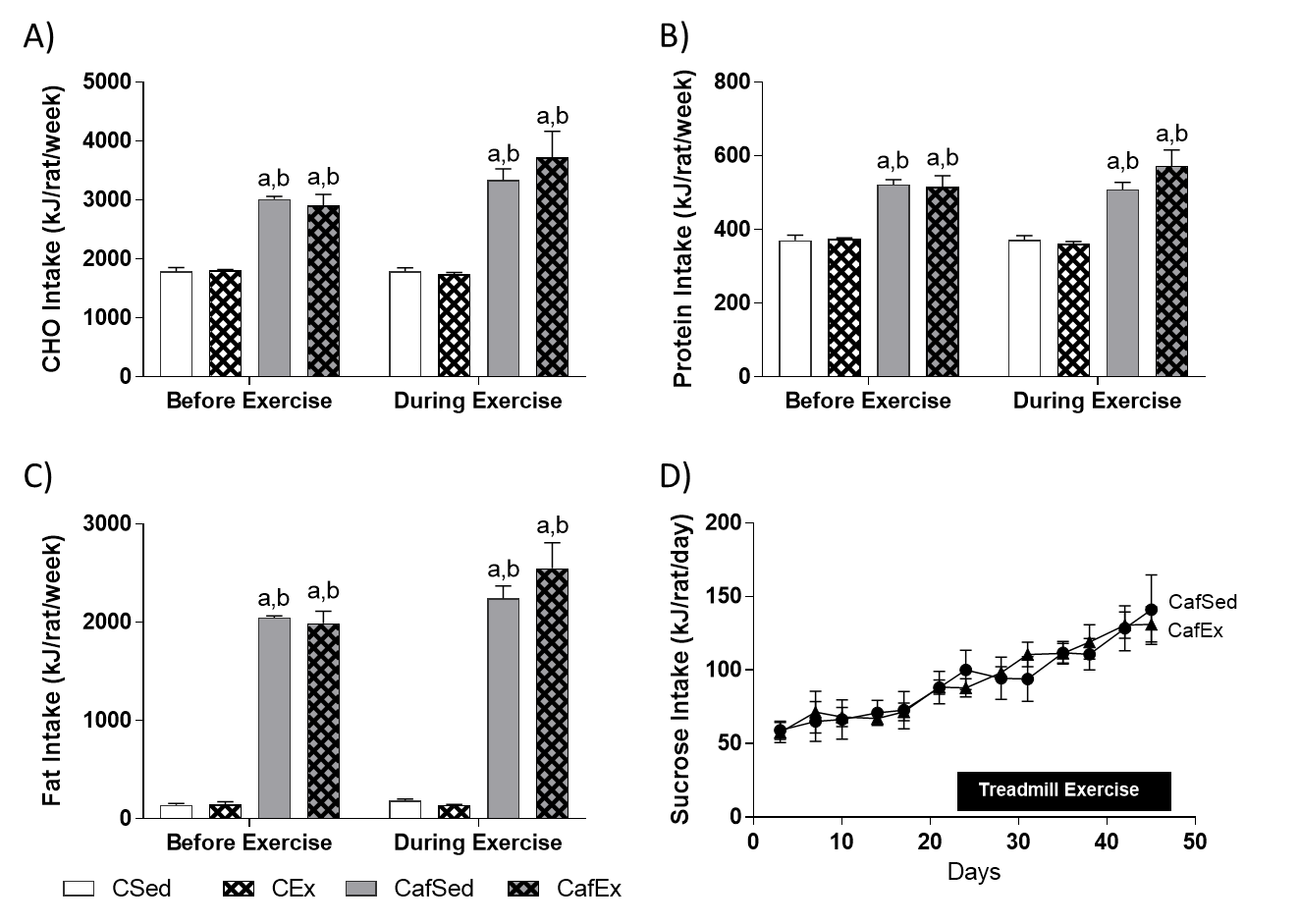


**Supplementary Figure 1.** Weekly macronutrient intake and sucrose intake over the study. Weekly (A) carbohydrate (CHO), (B) protein and (C) fat intake before and during the exercise intervention. (D) Average sucrose intake over the study. Data expressed as mean ± SEM; n=4 for cage data; data were analyzed by two-way ANOVA followed by Tukey-adjusted post-hoc testing.

^a^p<0.05 relative to CSed, ^b^p<0.05 relative to CEx

**Supplementary Figure 2.** Average composition of phyla between experimental groups. Data expressed as mean; n=12.

**Supplementary Table 2.** Distance-based linear modelling to determine contributions of metabolic and gene expression measures to the variance observed in microbiota composition at the OTU level

| **Marginal Model** | | | | | |
| --- | --- | --- | --- | --- | --- |
| Variable | SS(trace) | Pseudo-F | p-value | R^2^ |  |
| Diet | 18456 | 14.502 | 0.001 | 0.266 |  |
| Exercise | 1686.7 | 0.997 | 0.381 | 0.024 |  |
| Liver triglyceride concentration | 16013 | 12.007 | 0.001 | 0.231 |  |
| Plasma leptin | 15981 | 11.975 | 0.001 | 0.230 |  |
| Fat mass | 13329 | 9.516 | 0.001 | 0.192 |  |
| Plasma triglycerides | 11959 | 8.333 | 0.001 | 0.172 |  |
| Post-intervention weight gain | 10174 | 6.876 | 0.001 | 0.147 |  |
| Heart mass | 9499.3 | 6.348 | 0.001 | 0.137 |  |
| Lean mass | 8506.6 | 5.591 | 0.001 | 0.123 |  |
| Plasma insulin | 8764.2 | 5.785 | 0.001 | 0.126 |  |
| Retroperitoneal *Il6* | 3484.1 | 2.116 | 0.022 | 0.050 |  |
| Retroperitoneal IL-6 | 5796.8 | 3.648 | 0.002 | 0.084 |  |
| Hypothalamic *Crh* | 5617.7 | 3.525 | 0.004 | 0.081 |  |
| Hypothalamic *Npy* | 5496 | 3.442 | 0.004 | 0.079 |  |
| Hypothalamic *Cln5* | 5236.5 | 3.267 | 0.001 | 0.075 |  |
| Hypothalamic *Glut1* | 2304.3 | 1.375 | 0.123 | 0.033 |  |

Simultaneous distance-based linear modelling was used to investigate the unique contributions of diet, treadmill exercise and variables of biological relevance on the variance observed in the microbiome composition (captured by the Bray-Curtis similarity matrix at the OTU level). Simultaneous distance-based linear modelling interrogates the unique contribution of each predictor variable to the variance explained in the Bray-Curtis similarity matrix and significant predictors identified by simultaneous regression are shown in the first table; N=42-46
